# Recognizing Brain Pioneering as *Hard Work*: Strategies for Making Research Participation More Meaningful

**DOI:** 10.1002/hast.70047

**Published:** 2026-10-28

**Authors:** Erika Versalovic, Beatriz Mamede, Sara Goering, Eran Klein

**Keywords:** brain computer interface, BCI, neural device research, work, recognition, research participants, research ethics, bioethics

## Abstract

*Brain‐computer interface (BCI) research offers novel opportunities for new tools for mobility and communication for those limited by injury or illness. Despite this promise, current BCI research remains largely early stage and without direct therapeutic benefits for the trial participants with motor disabilities who invest extensive time and energy in research trials. In interviews our neuroethics research group conducted, participants in nontherapeutic BCI trials invoked job‐like or work‐like comparisons as they recounted the demandingness (long commutes, tedious tasks, and tiring hours) and rewards (skill building, satisfaction, and coworker‐like social relationships) of the trials. These comparisons, paired with the lack of therapeutic benefit, prompted our reflection on how participation in these long‐term, active‐engagement studies fits the conceptual framework of* meaningful work. *Examining trial participants’ testimonies through meaningful work's dimensions of excellence, social contribution, community, recognition, and agency reveals useful strategies for ensuring that the extensive time and effort trial participants invest in these trials is recognized, respected, and made meaningful*.

## Article

Research on implantable brain‐computer interfaces aims to create a new way of addressing a lack or loss of brain function. Through devices surgically implanted into the brain, researchers aim to restore or improve upon speech and movement capabilities. The public discussion of brain‐computer interface (BCI) research is driven by the BCI industry and often emphasizes its novel nature and encourages high expectations. BCI developers describe their devices and trials as “groundbreaking.”^1^ Blackrock Neurotech describes their work as “pioneering the future.”^2^ Neuralink announced that its implant currently in clinical trials will be named Telepathy.^3^ News coverage and researchers developing BCIs emphasize their potential not just to increase independence for users but also to change the way we interact with the world.

Media interviews of, publicity about, and the personal fame of some research participants all help to celebrate their roles as “brain pioneers.” Neuralink's first human participant, Noland Arbaugh, who received an implant in 2024, was interviewed over twenty‐four times in the first three months after his surgery.^4^ A YouTube video of Arbaugh playing chess using his BCI—a video in which he describes the experience as “like using the Force on the cursor”—has been viewed by millions of people.^5^ His journey continues to be tracked by a mass following on X.^6^ Similarly, in 2014, Juliano Pinto, a paraplegic man, captured the world's attention by using a BCI‐controlled robotic exoskeleton to kick a ball to start the World Cup tournament in Brazil.^7^ And nearly ten years before that, in 2005, Matthew Nagle gained fame as a participant in a Braingate study that allowed him to use an implanted BCI device to control a prosthetic arm.^8^ Public attention on these brain pioneers has celebrated the innovative nature of the research and suggests how exciting and rewarding participation in it can be. However, the headlines and news snippets often do not detail the extensive investment of time, effort, and resources required of research participants to help develop these technologies.

Our concern here is with the *work* that research participants put into developing such innovative technologies and with understanding their role within that research space, particularly when the demands of the trials are high and there is no intended direct therapeutic benefit.^9^ Are research participants appropriately understood simply as the subjects of research, who offer their willingness to undergo surgery and provide their data, or are they better understood as more actively engaged coworkers who deserve recognition for their contributions? We focus here on BCI research participants, although we offer our work as an exploratory case study that may be helpful for thinking about participation in other kinds of studies that are long term, have no direct therapeutic benefit, and can be quite demanding.

In our work as members of a research group focused on investigating ethical issues surrounding neural devices,^10^ we have interviewed and engaged with many participants in BCI studies. Our thinking about the work involved in BCI research participation grows out of those interactions.^11^ These research participants have been enrolled in nontherapeutic neural device trials that focus on basic proof of concept and on collecting data to better understand mechanisms that might one day be used to develop therapeutic interventions.^12^ Such research efforts stand in contrast to therapeutic studies, like those for deep‐brain stimulation or responsive neurostimulation, in which the research trial intervention could offer an immediate therapeutic benefit to participants.^13^ Nontherapeutic studies are often undertaken early in product development; in BCI research, for example, they might involve a cursor or robotic arm control paradigm that can be used only for very limited tasks and often only in a laboratory setting. They offer no potential clinical benefit to those who participate. They demand significant time and energy from participants without the promise of significant reward, particularly if the studies are longitudinal and require fairly intensive participant effort.

Despite the novel and cutting‐edge nature of the BCI research studies, many participants have described their experiences in them as surprisingly commonplace and job‐like, in terms of the tiring and demanding nature of the effort required; the sometimes humdrum, everyday nature of the experimental sessions; and the sporadic feelings of reward and accomplishment in the moments when their invested efforts paid off. This description of trial participation as feeling like a job or as a kind of work^14^ arose frequently in our interviews despite being unprompted. Participants recounted issues related to finding transportation to appointments, time spent on lengthy and tedious tasks, the mental focus required to perform the research activities, and colleague‐like research team dynamics. Since the onset of their conditions, many participants had been less able or unable to maintain employment or to participate in activities they had previously found socially meaningful, owing both to loss of motor control and to societal ableism that often led to social exclusion. The BCI trials provided access to forms of meaningful activities that many of them had been unable to find elsewhere.

Hearing their descriptions of research participation caused us to wonder, would it make sense to think of that activity as a kind of *work*? It certainly shares some contours of a part‐time job, in relation to scheduled time, energy commitment, and opportunities for developing social relationships, making contributions to something larger than oneself, and finding meaning or purpose. Yet viewing research participation as a form of work stands in contrast with prevailing models of research participation for nontherapeutic trials that typically frame it as altruistically driven volunteerism. In many clinical trials, the ideal participant is someone who wants to help future people and has no expectation of personal benefit. Research participants are also often thought of as inhabiting a relatively passive role. Considerations of what they are owed often focus on reimbursement (as outlined in study consent documents), adequate protections through an institutional review board or research ethics committee, and respectful stewardship of resulting project data.

These norms are ill fitting for early‐stage BCI trials. In most instances, BCI participants are not passively testing a new drug or device; instead, they must actively invest significant time, energy, and resources in the research projects. Participants are not volunteers who could be quickly and easily replaced by someone else if things don't work out. Instead, they become deeply integrated into research teams, and investment in their successful participation is significant.^15^ Given this unique positioning, the considerations of what they are owed would seem to merit more than merely satisfying basic requirements for research protection. But, given that research participants are not employees and that these activities occur within the setting of biomedical research, are their contributions reasonably understood as work? If so, what would such an understanding require ethically of these studies?

In this article, we use early‐stage BCI trials as a paradigmatic case to consider how research participation in long‐term, active engagement studies can fit the framework of meaningful work. “Work,” as we use it here, does not mean employment.^16^ Rather, “work” denotes an effortful activity that can be rewarding and from which one can derive meaning—and that, depending on the context and the way it is valued, can come with varying protections and forms of compensation.^17^ We argue that understanding research participation as a form of work expands the frame for ethical consideration beyond basic protection of participants to encompass active expressions of recognition and respect for the contributions of research participants.

To get here, we share the various ways participants themselves have spoken to us of their participation as job‐ or work‐like; present an overview of how research participation as work has been discussed within the research ethics literature; make a case for maximizing the goods of work for participants in nontherapeutic research trials; and, finally, offer strategies informed by participant testimony for helping participants attain these goods of work.

As a general orientation for the reader, the approach we undertake here is to revisit our previously conducted BCI participant interviews and view them through Anca Gheaus and Lisa Herzog's “Goods of Work” framework.^18^ Using this lens, we illustrate the ways such active and demanding research participation enables avenues for experiencing the goods of work—specifically, individual excellence, pursuing a purpose, finding community, achieving social recognition, and exercising autonomy. This approach enables us to highlight strategies that researchers can adopt to make research studies—not just BCI studies but also the active and demanding parts of clinical studies more broadly—more meaningful for research participants going forward. These strategies can serve as a toolbox for researchers seeking to identify what modes of increased engagement may be most appropriate and effective within their research context. Emphasizing the goods of work that participants can experience within research studies allows us to take seriously what participants report and want. It also advances the broader bioethical discussion about research participation as work.

## Neural Device Trials: “Like a regular job”

The context of long‐term implantable BCI trials is a paradigmatic example of the dynamics at play in demanding nontherapeutic studies. In early‐stage BCI trials, participants typically do not enroll with any real prospect for therapeutic benefit.^19^ The devices under study are often restricted to the laboratory setting and do not aid in regaining function for daily activities.^20^ Further, trials are demanding. They can require participants, and sometimes their care partners, to travel to lengthy research appointments multiple times a week, often over several years, to calibrate, test, and explore possible uses of the device.^21^ At the appointments, participants often put in significant effort, such that they report feeling exhausted and in need of rest after each session. In these ways, they are active contributors to the scientific endeavor.

We became aware of the demands of participation in neural device trials across multiple projects that our neuroethics research group conducted with participants in BCI trials.^22^ Interviews were semistructured, exploratory, and phenomenological in approach as they explored the lived experience of early‐stage BCI participants. (These are some of the questions we asked: What was it like to use a BCI device? How would you describe your experience in the trial? How did your participation in the trial affect your sense of control? Did you feel like your participation in the trial changed you in any way?) All interview participants were enrolled in early‐stage BCI development trials. As part of these studies, they underwent brain surgery to have electrodes implanted. After recovery, they went through extensive training (sometimes two to three times per week for one to two hours at a time) to learn to use the device (learning, for example, to use visual or motor imagery to control a computer cursor or robotic arm) and to help researchers fine‐tune it. Many of them stayed in the studies for three to five years, and some even longer than that.

Unprompted by us, multiple participants repeatedly used work‐like language to describe their participation in the research study. They did so both explicitly—using words such as “work,” “job,” and “coworker”—and implicitly—by describing job‐ or work‐like characteristics of participation, such as the demands of participation, their exhaustion, or the tedium of the activities (see table 1). Multiple participants described going to study appointments as akin to going to work or holding down a regular job. Participants also recounted the language that researchers in the lab used to refer to participants, such as sometimes calling them “coworkers.” One person directly compared research tasks to having a job without employment benefits. That individual noted the challenges of finding employment as someone with quadriplegia and their inability to qualify for disability benefits due to not being employed at the time of their accident.

**Table 1:** Participant Depictions of BCI Trial Participation as Work‐Like

**Trial tasks directly described as “work” or a “job” or participants characterized as workers**
“I felt like it was my job. Even my daughter would ask for something and I'd say, ‘Well, I have work tomorrow’” (Participant 3).* “‘I don't look at you as a lab rat. You're our coworker. You're our colleague.’ It was such a nice sentiment, and that's how they all treated me” (Participant 4). “Basically, it's a job” (Participant 5).
**Descriptions of job‐like aspects of participation, such as exhaustion, demandingness, and tedium**
“I knew it was going to hurt, the surgery, but I don't think they knew this either—how tired I would be after a session, because by the time I would get home, it was an hour ride to get home, and then I'd naturally get put in bed, and I'd go to sleep for a couple hours just because I was tired from working” (Participant 3). “Like a regular job, some sort of regular job that you're getting up in the morning and you commute to and you're there for a while, and because it's definitely not all fun and sometimes it's really boring” (Participant 5). “Well, when I had to travel to and from the lab, I would spend a good six hours away from home—because driving through traffic, whatnot. And not to mention, physically, my pressure ulcers got worse” (Participant 2).

* The parenthetical information in the table indicates participant numbers that we assigned across studies.

Implicit mentions of aspects of work also arose across the interviews. Multiple participants emphasized how tiring, and occasionally boring, trial sessions could be. They recounted being surprised by the demandingness of trial tasks and needing to rest after experimental sessions. Participants also mentioned the tedium and the burden of the commute time on them and their care partners to get to and from experimental sessions.

The work‐like language in our studies extended to the social contribution that can be a part of work. One former health care worker noted that participation in BCI research “gave me the opportunity to continue down that path of being able to be in a position to give back or to give the talent” (Participant 2). Language of social contribution parallels testimonies reported by Jox Kogel and colleagues in their interview study with BCI study participants. After exiting the BCI study, one of their interviewees expressed, “What I miss even more is having the job. I went to the office three days a week. I had co‐workers, people I worked with and became close to. I miss the camaraderie of working with the group. I miss having a place to go and something to do every day.”^23^ Across research studies, research participation seems to provide a sense of purpose, a rewarding work community, and the camaraderie of feeling like coworkers.

These parallels with characteristics of typical jobs led us to consider the implications of taking this rhetoric seriously. What might using a work frame mean for understanding best practices in the design and implementation of long‐term nontherapeutic research studies? Institutional and political contexts necessarily shape this inquiry. For instance, ethical norms regarding compensation for research participation and concerns about undue inducement may set some limits, as will political realities regarding earning caps for people receiving Social Security Disability Insurance, at least in the U.S. context.^24^ Rather than arguing explicitly for a wholesale shift toward work‐like financial compensation for study participants, we consider more holistically the kinds of benefits that can follow from recognizing participants’ efforts as work. Identifying potential goods of research participation can help address participant desires for meaning, purpose, and acknowledgment of their contributions.

## Getting to Work within the Research Context

Emily Largent and Holly Fernandez Lynch,^25^ among others,^26^ have argued against research practices that inadvertently risk exacerbating inequities within health research. Pushing back against medical research exceptionalism—the general view that clinical research is meaningfully different from settings in which people accept similar degrees of risks and thus merits more stringent regulations and protections—Largent and Lynch take particular aim at practices of low compensation within clinical trials. In their account, the lack of guidelines surrounding when payment thresholds are “appropriate,” combined with fears regarding sufficiently protecting participants, lead to overly conservative approaches to compensation. The resulting meager compensation norms risk unfairly burdening participants (for example, by leaving them unable to offset the day's wages forfeited by prioritizing a study appointment) and undervaluing their contributions. Largent and Lynch's view can be seen as an entry point to a broader conversation about research participation as work.

Similarly, Matt Lamkin and Carl Elliott push back against the low compensation norms and lack of sufficient research protections for research participants, particularly those in phase 1 clinical trials.^27^ They advocate for designing informed consent agreements for research participation to be similar in ways to an employment contract that guarantees the employed person certain worker rights. They recommend increased compensation alongside “worker protections” under which people can seek remediation if they are harmed during a trial.

These efforts are broadly consonant with a strain of bioethics scholarship that has advocated for research trial participation to emulate “decent work” standards.^28^ The decent‐work doctrine is grounded on the International Labour Organization's 2008 Decent Work Agenda.^29^ In this domain, work is decent when it ensures safety, equal treatment, social protections such as proper health care or worker's compensation, and fair pay.^30^ These standards focus on the minimum protections workers should be guaranteed for their investment in the work.

Interestingly, this scholarship on research participation as work has tended to limit its focus to studies that either have relatively low demand in terms of time or effort or offer a potential therapeutic benefit. Regarding the first, the research participants that scholars have in mind tend to be those who are “paid to endure.”^31^ While they may need to be monitored for side effects, their active contribution to a study is minimal, consisting mainly in such steps as getting to appointments on time and following directions. Some of these participants see themselves as working as “guinea pigs” to earn money.^32^ Second, Largent and Lynch note,^33^ there are patients in later‐stage clinical trials who hope for clinical benefit, even if they cannot rightly expect it. They take a drug or treatment and then undergo observation and testing for safety and effectiveness; as with participants in the first category, their role—while notable and praiseworthy, given the risks they take on—is relatively passive. In other words, paradigmatic cases of “research as work” that focus on ensuring minimum decency tend both to be short in duration and to require only modest effort.

Higher compensation for time and forgone opportunities, as well as better protections for any harms experienced in the course of the study, helps to recognize the role of the research participants. For these cases, the decent‐work frame effectively draws attention to potential exploitation and ways to support participants, given the risks they take on behalf of research.

However, the decent‐work frame leaves a notable gap unaddressed: what is owed to participants in more active and demanding studies, particularly when there is no chance for therapeutic benefit? Higher compensation alone may not be feasible (given political and policy constraints related to Social Security Disability Insurance), and basic workplace protections, while important, may not be enough to recognize the active contributions of and the burdens taken on by participants.

In other words, ensuring that the work of research participation is decent does not fully recognize that participants in more time‐ and energy‐intensive studies, like implantable BCI trials, may desire and often deserve more than basic protections. When participants’ investment of effort and time (in addition to risk assumption and vulnerability) increases with no potential for therapeutic benefit within the study, the need to consider other kinds of benefits that participants may derive from the study grows.

Instead of focusing primarily on how to model BCI research participation as decent work, then, we suggest going further to focus on the goods of work and what makes work meaningful. While compensation can be one of these goods, people work for many reasons, and compensation is not the only one—and is sometimes not the primary one. Work is also about investment of time, energy, and attention directed at something that is larger than oneself and the rewards that come from accomplishment and mattering. What are the kinds of goods that emerge through work?

## Research Participation as Meaningful Work

Recent scholarship has focused on the concept of work, including the role that it does or can play in people's lives. This scholarship spans a range of disciplines, from psychology^34^ to business management^35^ to philosophy.^36^ In particular, the connection between work and meaning has drawn attention.^37^ Although no consensus definition of “meaningful work” has yet emerged across this scholarship, how a worker assesses, evaluates, or esteems the significance and value of their work is often deemed central.^38^

One framework for making sense of meaningful work focuses on the goods of work: excellence, experiencing community, social contribution, social recognition, and autonomy. This framework has found little uptake (with a few exceptions^39^) in the context of research ethics, perhaps owing to the ways that less demanding research studies (such as those involving “guinea pigging”) have often been used as the guiding examples to elucidate the “work” of research trials.^40^ Here, we invoke a modified version of Gheaus and Herzog's model of meaningful work to explore the types of benefits participants sometimes receive through the BCI trials. The lens it provides helps highlight the ways that participation in these more demanding, long‐term trials go far beyond the archetype of the more passive participant volunteer.

In this meaningful‐work framework, we focus on individuals’ perceptions of their work as both significant and intrinsically rewarding.^41^ Meaningful work, as it is often defined in the broader literature, has consequences that are mutually beneficial for individual workers and the groups within which individuals perform that work—namely, personal and professional engagement, general satisfaction, and a positive psychological flourishing.^42^ In addition, supporting meaningfulness has noticeable impacts on people's motivations, energy, and personal fulfillment. Some authors even conceptualize meaningful work as a fundamental human need.^43^

Meaningful‐work theory is not fully separate from, but rather builds on, the groundwork of decent work.^44^ The concept of meaningful work does not, by itself, remove the possibility of unpleasant emotions and negative experiences related to work.^45^ Pursuing difficult‐to‐achieve goals through one's work can be highly rewarding and meaningful. In our context, BCI research participants recounted their feelings of fatigue and exhaustion in tandem with reflecting on their sense of purpose during the research. Hard work, in service of a valued goal, can feel rewarding. Meaningless work, by contrast, is commonly associated with symptoms of “burnout, apathy and detachment.”^46^

While we start with the four goods of work identified by Gheaus and Herzog, we propose amending their framework by adding autonomy, as suggested by Jilles Smids, Sven Nyholm, and Hannah Berkers.^47^ Together, these goods comprise the sense of meaningfulness that participants can derive in these studies: excellence, social contribution, the experience of being in community, social recognition, and autonomy. By considering the ways these five elements arose in our interviews, we highlight the potential for meaningfulness and purpose in these studies. Different goods arose for different participants in different studies. The excerpts we highlight here illustrate instances of how these goods were experienced by participants and provide an entryway for researchers to consider how to strengthen avenues for participants to attain them.

The first aspect of meaningful work is the possibility of attaining excellence. This aspect appears in the literature in many forms, overlapping with the idea of self‐development or skill exercising.^48^ The intent is to foster a space to cultivate new abilities and improve one's performance or personal skills. For the BCI users we interviewed, learning to use a robotic arm required extensive training, troubleshooting, and practice. Participants recounted the need for persistent effort in order to see improvement, with one explaining, “They [the researchers] had already written in an offset that I had to truly learn before I could be successful on that task and that took a while” (Participant 3). One participant described the kind of mental flow state that had to be honed over time for them to be successful on trial tasks: “I just say, ‘Zen mode.’ It's less focused on one particular thing. I'm not really focused on looking at a certain target or anything…. When I'm just like that, I can overcome things like the [robotic arm] biases” (Participant 5). Participants who put in the work to learn to use a BCI skillfully were rewarded with a feeling of achieving excellence.

Social contribution is also a part of meaningful work. Working for something larger than oneself helps one to forge a sense of mattering in the world. Altruistic aims are a common reason for participants to join a research trial, with the goal of advancing scientific progress and assisting future people impacted by similar disabilities, conditions, or limitations. The will to actively make a difference to bring about a greater good is a key initial motivation for wanting to be a part of research projects, and it's a driving force to push forward on hard days. As one of our participants proclaimed, “Prior to that, it was always people helping me, and I wasn't able to help anybody physically. The chance to actually be able to help was an overwhelming factor” (Participant 1). Research participants appreciate the opportunity to do their part in creating a larger social good.

For many theorists, meaningful work also involves experiencing community. This element calls out the common desire to be part of a project or, as Gheaus and Herzog discuss, to participate in collective agency.^49^ As described by our participants, their peer‐like social relationships and community with the research team went far beyond the scope of a helpful volunteer.^50^ The depth of this involvement with the research team hearkens back to James Anderson and Charles Weijer's call to treat subjects as “partners in research”: feelings of cooperation and care between researchers and trial participants lead to increased excitement and satisfaction in the activity.^51^ In the BCI context, unlike in research contexts where researchers and participants may stand in a relatively rigid hierarchy with respect to each other, participants often feel a close camaraderie with the researchers. Not only do participants state that they feel “part of a team,” but they sometimes also see themselves as friends or even as members of a family with the research team: “[I]t's family. And I believe that after I'm done with this study, I will still have a relationship with the members of a team because individually they all look after my best interests” (Participant 2). BCI research participants may start to feel that they are a valued part of the research group as a whole, rather than playing a more passive, peripheral role. This participant went on to say, “It's the opportunity for me to get out there again in the public. It's a social benefit for me. And with my particular study, I'm able to interact with other people who have a shared interest in what we're going through in this trial, so they value my input.” Similarly, through the research environment, participants may connect with other research participants like themselves, people who have comparable disabilities, similarly demanding study commitments, and novel experiences in using the technology (for an example of a forum in which participants form these connections, see the BCI Pioneers Coalition^52^). These relationships, initiated within the space of the research, develop into a community of support and shared aims.

Another element of meaningful work is social recognition. Gaining social recognition depends on an external acknowledgment or social credit that enhances the participant's sense of accomplishment and, therefore, respect for their work. Although participants may be making a social contribution through their participation, that contribution may or may not be recognized. For participants, receiving that recognition can improve their well‐being and their sense that the effort they invested was worth it. As Andreas Veltman observes, objectively meaningful work can be built not only by the development and exercising of one's capabilities as key components of excellence but “especially insofar as this expression meets with recognition and esteem,” which opens space for individual growth.^53^ In the BCI trial context, recognition can come from the sense of being a pioneer in the field, given the novelty of the technology, as well as the possible future uses of the technology to enhance functionality for disabled people.^54^ BCI users commented positively on this sense of being noticed and acknowledged, with one of the participants reporting, “Even now, I go to my barber and the first thing he asks is, ‘What have you guys done now?’ There's genuine interest” (Participant 2). Another recounted, “I got to go to the conference and be around all these scientists and talk about the brain array, and it was a great experience for me” (Participant 3). Of course, social recognition need not be so broad; it could be local social recognition, as when the rest of the research team offers appreciation for the participant's achievements in any successful experimental session.

The last component of meaningful work within our framework is autonomy. Autonomy is frequently cited across the meaningful‐work literature as an important good to strive toward.^55^ In the workplace, it can take the form of individuals’ being afforded freedom to adjust or shape their tasks or to make independent decisions that demonstrate control within their roles.^56^ In the BCI context, where research participants and researchers mostly work in tandem, participant autonomy takes the form of researchers’ being responsive to suggestions from participants during training sessions. A participant recounted, “Sometimes they would set the gain too high, and I felt like I would share and tell them immediately, ‘Hey, lower the gain because, if not, I don't need to be here.’ … I was there to work, and I wanted to make sure it was me doing the task and not the help of the computer accomplishing it” (Participant 1).

We don't mean to imply that our BCI participants always experienced the goods of work or always experienced them fully. In fact, some participants expressed views that suggest that they missed out on or were kept from the goods of work, and they shared their frustrations. Three examples stand out.

First, participants mentioned cases in which they were not able to exercise their autonomy within a study, where their input or opinion could have been solicited but was not. One participant recounted frustration with someone whom they took to be a disrespectful research team member: “They were hiring people that I would spend four, five hours a day …. Some of these people I didn't want to be around…. I bring a ton of money to a project and you can't even consult me if this person is someone I want to be around?” (Participant 1).

Second, participants expressed frustration with not getting recognition for their contributions or even receiving information on the results of their contributions. One person reflected on the need for follow‐up regarding research products and for avenues to access them: “I might not even know they actually finished writing the paper, and it got published somewhere. And those are not accessible easily if you are not in the know. I can just ask to get sent a copy, but if I'm searching online, I can find the paper, but you have to be in one of the clubs that you're paying money for to access the papers…. It just basically goes along the same lines of don't ghost the person” (Participant 5).

Third, participants expressed a sense of unfairness about how much a device company benefited from their participation relative to the small levels of compensation or reimbursement they received for participation: “Because the project is receiving multimillion dollar…. [W]e're talking over 150 million‐dollar donations, and I'm over here getting 50 dollars for my fucking time when just to come [to the lab] it would take several hours. I'm helping to make careers. These people are going to be recognized for the rest of their lives for what they accomplished, what I'm helping them accomplish, and they're not recognizing me at all” (Participant 1).

Building avenues for participants to receive the goods of their work through these research trials helps to protect against these frustrations and to recognize their efforts. We do not take these goods to be an exhaustive list of the types of goods that people can derive through such studies. We offer them, rather, as a starting framework for considering the kinds of strategies that can help strengthen research participant experiences. Examining each aspect in turn illuminates the range of ways research participants’ contributions can be recognized in these early‐stage research projects.

## Strategies for Making BCI Work More Meaningful

The meaningful‐work framework goes beyond being a tool for interpretation; it clarifies pathways for strengthening participant engagement and recognition. Here, we consider each good of work in turn to offer practical recommendations for recognizing and honoring participant contributions in research studies. Again, we take the context of early‐stage BCI trials as a paradigmatic example of demanding research studies, with respect both to duration and to the time and energy. We spotlight the strategies for honoring participants’ work that have been employed by some research teams in this context. We propose that the actionable strategies these studies illuminate can inform practices in similarly demanding studies and can extend in varying degrees to less intensive studies. The strategies build out a toolbox that research teams can draw on both inside and outside the BCI context.

***Attaining excellence***. Strategies that can help honor the effort that goes into attaining excellence within the trial parameters include being responsive to the energy expenditure of trial tasks, recognizing the skill building that occurs within the study, and constructing personally meaningful project tasks when possible. Examples of these strategies include offering frequent breaks, checking in about participants’ capacity for various tasks, marking milestones in achieving new study skills, or adapting certain study tools for at‐home use to extend the applicability of the skills gained through study participation.

***Social contribution***. People often enroll in research trials as a way to be part of something larger than themselves and out of a desire to give back. The sense of contributing to scientific progress can be particularly difficult in early‐stage trials, where results may be less definitive and trial tasks more exploratory. Research teams can scaffold participants’ social contribution through providing additional ways of giving back (such as connecting them with prospective participants), considering ways to honor and recognize participant motivations (such as by returning research results and providing ongoing research updates), and sharing information about a study's progress with participants’ family members.

***Experiencing community***. Unlike in some forms of research participation—such as what Erik Malmqvist describes as the more transactional dynamics between researchers and healthy participants in phase I trials^57^—in the case of intensive, long‐term research studies, participants often feel that they are a key part of the research team. The new social relationships that emerge through study participation with the researchers or other participants can become a meaningful form of community, particularly given the social isolation that some experience. In addition, they often get to know the research team well, given the amount of time they spend together. Research teams can help foster community among team members and participants by building in time for informal check‐ins and treating participants as coworkers and peers when appropriate. Work communities do not last forever, but recognizing transitions in and out of communities can help people feel more valued for their roles in them.

***Gaining social recognition***. Ways of giving social recognition for participants’ work can operate at multiple levels: within the study team, within more limited professional spaces, or, for those desiring this, across the broader public. These strategies could include celebrating the achievement of particular study tasks, inviting participants to speak at conferences, or connecting them with a journalist who may be interested in writing about a novel technology. Of the strategies we highlight, those for helping participants gain social recognition are particularly likely to require high variability across participants.

While many research norms default to an assumption that preserving anonymity is a top concern, participants sometimes want to be more broadly recognized for their particular contributions within a project. We encourage research teams to engage in conversations about disclosure preferences with each participant to help open appropriate avenues for this form of potential benefit. Such conversations are made more manageable by the small number of participants in studies like these.

***Autonomy***. Research trials can enhance avenues for empowering participants both within and beyond the trial. The constraints many research participants with disabilities face due to societal ableism and physical constraints heighten the importance of finding pathways for supporting participant agency in research. Approaches for respecting participant autonomy include welcoming participant feedback on research practices (for example, cocreating data‐sharing practices), allowing participants to voice concerns when conflicts arise (for example, requesting space from a researcher who made a racist or sexist comment), and increasing compensation levels.

Although we have parsed these goods of work into different categories with different tools for promoting them, we recognize that they overlap in practice. For instance, offering opportunities for recognition, such as by presenting at a professional conference, both honors the skills built through trial tasks and enables social recognition. Similarly, respecting participants’ motivations to be part of the trials honors their social contribution in a way that also respects their individual autonomy within the constraints of the trial. An example would be returning study results to those who were motivated to participate out of a desire to contribute to scientific progress.

Further, just as meaningfulness varies person to person, these strategies are meant to be tailored to each trial context and participant. These strategies are aimed at the plurality of benefits that participants might receive through research participation, but whether employing more strategies is desirable will depend on what kinds of benefits are most valuable to participants in each trial context.

Finally, we recognize that attending to the meaningfulness of research participation cannot be wholly bracketed off from questions of compensation. As recounted by multiple participants, societal ableism restricts the employment opportunities for those with disabilities. Participants described the ways study stipends fell short of covering caregiver costs and transportation expenses. Given the extent to which participation in studies can mirror employed work, we encourage research teams to increase compensation levels when possible while attending to regulations that may limit them. Although compensation is only one piece of the broader picture of the plurality of benefits that come through participants’ research labor, it is a particularly important piece.

## Reciprocal Benefits

Viewing participation in demanding research trials like nontherapeutic BCI studies through the lens of meaningful work highlights the importance of honoring the effort participants invest in the research and aligning research practices with participant needs and interests. These trials ask a great deal from participants and their care partners—time, energy, potential risks—without a chance of therapeutic benefit. For many participants, the trials offer an opportunity to gain not only a sense of purpose but also meaningful forms of engagement, recognition, and social connection that felt difficult to find following the onset of their conditions.

By considering five goods of meaningful work in turn—attaining excellence, making a social contribution, experiencing community, gaining social recognition, exercising autonomy—we can better identify strategies to strengthen the kinds of benefits that participants attain through research participation. Building additional avenues for these valuable aspects of participation helps honor the time, effort, and risks participants invest in research. Of course, making work more meaningful will not protect against all problems participants face in a trial; just ask an underpaid research intern. Making work meaningful must be pursued in tandem with providing more minimal research protections, such as the ability to seek redress for harms and reimbursement for costs.

Researchers may be wary of having additional expectations placed on them, but making work meaningful for participants can come with reciprocal benefits for the broader research team. Researchers both get the opportunity to learn from research participants in ways that lead to more impactful research and feel reassured that they are not using participants as means to their own ends. Taken together, the strategies described above open ways to empower research participants by ensuring that they are valued contributors to scientific progress. Identifying participants’ contributions as work, and striving to make it meaningful work, are ways to recognize their critical role in this field and will help the field develop responsibly.

## Acknowledgment

This article is part of a series, Neuroscience and Society, whose development by The Hastings Center for Bioethics is funded by the Dana Foundation.

The authors are grateful to their reviewers and to the University of Washington neuroethics research group for their helpful discussions of the ideas presented here. This work was supported by National Institutes of Health award R01MH130457.
